# Self-Taught Learning Based on Sparse Autoencoder for E-Nose in Wound Infection Detection

**DOI:** 10.3390/s17102279

**Published:** 2017-10-07

**Authors:** Peilin He, Pengfei Jia, Siqi Qiao, Shukai Duan

**Affiliations:** College of Electronic and Information Engineering, Southwest University, Chongqing 400715, China; qaz321123@email.swu.edu.cn (P.H.); i47sir@126.com (S.Q.); duansk@swu.edu.cn (S.D.)

**Keywords:** electronic nose, self-taught learning, sparse autoencoder, wound infection

## Abstract

For an electronic nose (E-nose) in wound infection distinguishing, traditional learning methods have always needed large quantities of labeled wound infection samples, which are both limited and expensive; thus, we introduce self-taught learning combined with sparse autoencoder and radial basis function (RBF) into the field. Self-taught learning is a kind of transfer learning that can transfer knowledge from other fields to target fields, can solve such problems that labeled data (target fields) and unlabeled data (other fields) do not share the same class labels, even if they are from entirely different distribution. In our paper, we obtain numerous cheap unlabeled pollutant gas samples (benzene, formaldehyde, acetone and ethylalcohol); however, labeled wound infection samples are hard to gain. Thus, we pose self-taught learning to utilize these gas samples, obtaining a basis vector θ. Then, using the basis vector θ, we reconstruct the new representation of wound infection samples under sparsity constraint, which is the input of classifiers. We compare RBF with partial least squares discriminant analysis (PLSDA), and reach a conclusion that the performance of RBF is superior to others. We also change the dimension of our data set and the quantity of unlabeled data to search the input matrix that produces the highest accuracy.

## 1. Introduction

Electronic nose (E-nose), a device composed of a sensor array and an artificial intelligence algorithm, has been successfully used in many fields. It is able to deal with a multitude of problems efficiently, such as food analysis [1,2,3,4], disease diagnosis [5,6,7,8], environment control [9,10], etc.

Traditional methods for a doctor to diagnose the type of wound infection usually require observing features of the plaie and take a long time to analyse the patient’s blood, urine and other aspects, delaying the best time for treatment. In particular, with the development of medical technology as well as higher requirements on disease detection speed and accuracy, the E-nose has great prospects in disease diagnosis. Our previous work has proved that the E-nose can be used to distinguish the classes of wound infections through their special odor [11,12,13,14].

In practice, however, if we want to get an E-nose that can distinguish wound infections efficiently and accurately, quantities of wound infection samples are needed to train the classifier, which would cost a lot of money. Such experimental infection samples are not that easy to obtain, let alone labeled wound infection samples. While our wound infection samples are limited, there are some other unlabeled pollutant gas samples which are numerous and obviously easier to obtain, for a lower cost. If we ignore the usage of these samples in other fields, it can lead to waste. To take advantage of them, we introduce transfer learning in our paper.

Transfer learning is the ability to transfer knowledge from one field to other fields, and these fields can share different labels, which distinguish it from traditional machine learning techniques. That is to say, through transfer learning, we can use some samples from other fields to make up the lack of wound infection samples in our field. Thus, in this paper, we purchase inexpensive chemical solutions to obtain four types of pollutant gases, and can, as a result, get thousands of unlabeled samples through this approach, at low cost.

These unlabeled gas samples are introduced to cope with the lack of labeled wound infection samples, and an enhanced quantum-behaved particle swarm optimization (EQPSO) [15,16] is proposed to improve the performance of classifiers. In the machine learning field, there are some classical algorithms that can roughly be placed in two categories [17]: supervised learning, which concerns obtaining its classifier based on labeled data; and unsupervised learning, which is concerned with obtaining its classifier from unlabeled data. Unavoidably, however, both methods have significant shortcomings: supervised learning needs a large amount of labeled data, while unsupervised learning classifies samples by their different distribution, which makes the accuracy far lower than that of supervised learning. Therefore, semi-supervised learning, a combination of these two types learning framework, is widely adopted in practical application and improves the generalization ability of model. It broadens the range of data set, but because semi-supervised learning is typically based on the assumption that labeled data and unlabeled data can be tagged with the same labels, a new group of machine learning is put forward, which is called “self-taught learning”.

Self-taught learning [18,19] is a new machine learning framework and also a type of transfer learning, corresponding to human learning, using unlabeled data in supervised classification tasks. What distinguishes self-taught learning from other learning methods is that self-taught learning can solve such problems as the fact that labeled data and unlabeled data do not share the same class labels, that they may be from entirely different distributions, or that the labeled data might be far less than unlabeled data.

In recent years, self-taught learning has undergone considerable development in many fields [20,21,22]. In self-taught learning, we construct basis vectors from the unlabeled data. In turn, these basis vectors are used to rebuild input representation, converting training data into representations related to unlabeled data. These new representations are programmed into the classification task and significantly improve the performance of the E-nose. In the algorithm, the most significant step is to contrast basis vectors from the unlabeled data and to rebuild new representations. To rebuild new representations, we take advantage of the neural network and apply the sparsity constraint, which makes the representation of each layer sparse (most of nodes become zero).

However, this is yet to be applied in the field of E-nose for the purpose of distinguishing the label information of wound infection data. In this paper, self-taught learning is proposed to perfect the accuracy of classification. In the rest of this paper, we first describe details of the material and odor sampling experiments in Section 2, then the self-taught learning framework is elaborated on in Section 3. In Section 4, we apply some classical classification algorithms and compare their results, such as partial least squares discriminant analysis (PLSDA) and radial basis function (RBF) [23,24].

## 2. Experiments and Data Preprocessing

### 2.1. E-Nose System and Experimental Setup

**Figure 1:**
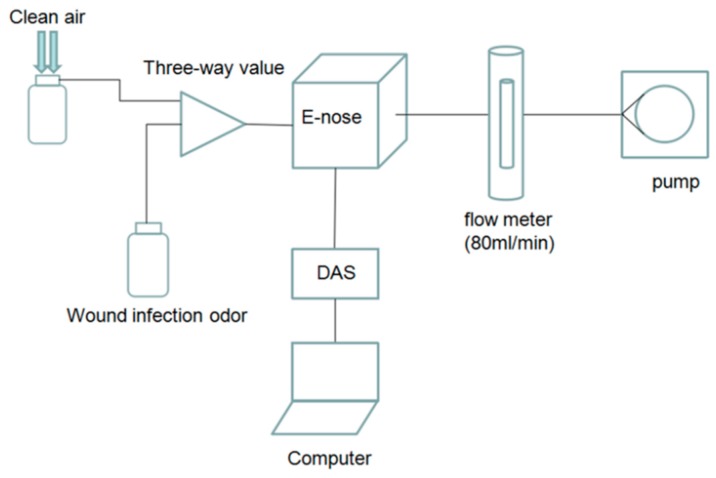
Schematic diagram of the experimental system.

**Figure 2:**
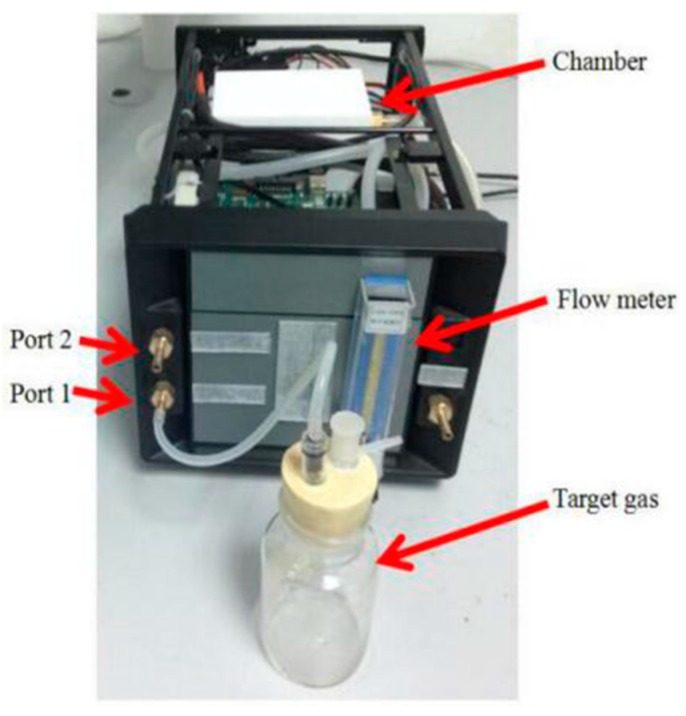
Experimental setup.

The labeled wound infection data set and unlabeled gas data set are needed in our project. An E-nose system is used to prepare the data set. In constructing the system, we employ an E-nose, a data acquisition system (DAS), a pump, a rotor flow meter, a three way value, a filter, glass bottles and a computer. The schematic diagram of the experimental system is shown in Figure 1 and the experimental setup is shown in Figure 2.

When air passes the filter, the air is purified. The flow meter controls the rate of the gas, making it keep at 80 mL/min, and at the same time the pump provides energy for the gas flow. The response signals processed by E-nose will be sampled and saved in a computer via the DAS, which is 32-channel and 14-bit high precision. The sampling frequency is set at 1 Hz. According to the metabolites of pathogens and the response characteristics of gas sensors, a sensor array composed of six sensors is employed to collect the response curve of wound infections and pollutant gases, and response characteristics of gas sensors are shown in Table 1.

Each sampling experiment is composed of the following three steps:

Step 1: the sensors are exposed to clean air for 3 min;
Step 2: the gas stream containing VOCs of the wound passes over the sensor array for 5 min;
Step 3: the sensors are exposed to clean air again for 15 min.

The sample interval between two experiments is 5 min.

### 2.2. Experiments and Sampling

Twenty Sparague-Dawley (SD) male rats are used in the experiment in this paper to prepare the labeled data set. These rats are divided into four groups averagely:

1. Wounded but uninfected (the control group);
2. Infected with *P. aeruginosa*;
3. Infected with *E. coli*;
4. Infected with *S. aureus*.

All rats are healthy and in similar condition, and each type has five rats, respectively. Every rat has a 1 cm long wound in the right hind leg, and pathogens are injected into the wound in accordance with their group. The metabolites of three pathogens are shown in Table 2. A total of 20 sampling data are collected for each kind of rat, that is to say, there exist 80 labeled wound infection samples in our project.

And four kinds of pollutant gases including benzene (C_6_H_6_), formaldehyde (CH_2_O), acetone (C_3_H_6_O), ethylalcohol (C_2_H_5_) are sampled as the unlabeled data set.

Before the sampling experiments, we firstly set the temperature and humidity of the chamber as 25 °C and 40%. Then we began the gas sampling experiments. Because some of the gases are liquid, a decompression device is used to convert the liquid to the gas phase. We took advantage of the same setup and sensor array used for wound infections to get the pollutant gases’ data set and each sampling experiment was strictly executed according to the 3 steps in Section 2.1. In total, we collected 2664 samples of pollutant gases.

To get the real concentration of each gas in the chamber, we extract each gas from the chamber and import it into the gas bag. Then spectrophotometric method is employed to get the concentration of formaldehyde, and the concentration of benzene, acetone and ethylalcohol is determined by gas chromatography (GC). The real concentration of three gas is shown in Table 3. For the four gas, there are 12, 11, 12 and 21 concentration points, respectively, and 12 sampling experiments are made on each concentration point.

In most experiments, we only applied 652 samples and detailed information is shown at Table 4.

**Figure 3:**
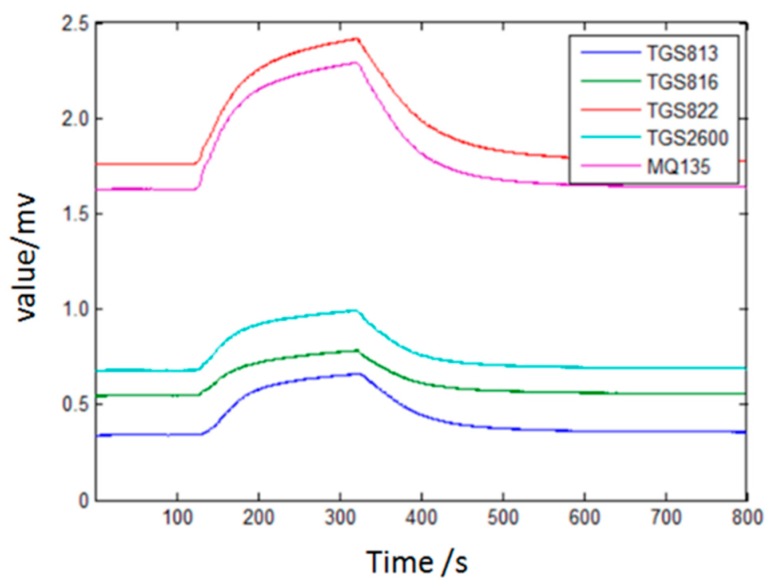
Response curve of the sensor array of *S. aureus* (one of the wound infections).

Figure 3 illustrates the sensor response process when the sensor array is exposed to four types wound infection odor. It is clear that each curve has a rise when the target gas passes over the sensor array.

### 2.3. Data Preprocessing

To find which feature matrix can obtain the best performance, we extract five different features to construct our data set. These features include:

(1) Maximum value of steady-state response;
(2) Maximum slope of rising edge;
(3) Maximum slope of falling edge;
(4) Integral;
(5) Wavelet transform.

Except for wavelet transform, all other features are easy to understand. Wavelet transform is a kind of local transformation of time and frequency domain, which can efficiently fetch information from the signal, and it also has been used in the E-nose before [25]. It inherited and developed the localization of the short time Fourier transform (STFT), at the same time overcoming the shortcomings, such as window size, which does not vary with frequency change. Wavelet transform can provide a time–frequency window that changes with the frequency, and is an ideal signal time–frequency analysis and processing tool.

After feature extraction, the labeled data set and unlabeled data set share the same dimension. We use the five features to construct original feature matrices, each row is a feature, and each column is a sensor selected from the 5 sensors. Then, we transform this n×n matrix into $1\times n^{2}$ matrix, building the data set $x\in R^{n^{2}}$, and $n^{2}$ represents the dimension of x. To get training sample$\;x_{l}$, we randomly pick 15 samples from each wound infection sample (in total, 20 samples in each wound infection type), then we have 60 training samples$\;x_{l}$ and 20 test samples$\;x_{t}$. We also construct a data set comprised of all unlabeled samples $x_{u}$ to train a basis θ, and $\;x_{u}$ contains four types of gas. In this paper, n is set as 3, 4, 5 to find which n is most efficient to improve the performance of the E-nose.

## 3. Self-Taught Learning

In this paper, we apply the self-taught learning paradigm with sparse autoencoder and classification algorithms (like RBF) to build a classifier for distinguishing different types of wound infection.

Suppose there is a labeled data set of m samples $\left\{\left(x_{l}^{\left(1\right)},y^{\left(1\right)}\right),\left(x_{l}^{\left(2\right)},y^{\left(2\right)}\right),\ldots ,\left(x_{l}^{\left(m\right)},y^{\left(m\right)}\right)\right\}$. Each $x_{l}^{\left(i\right)}\in R^{n^{2}}$ denotes an original input feature vector. Each $y^{\left(i\right)}\in \left\{1,2,\ldots ,C\right\}$ denotes corresponding class label. Additionally, we assume there are k unlabeled samples $x_{u}^{\left(1\right)},x_{u}^{\left(2\right)},\ldots ,x_{u}^{\left(k\right)}\in R^{n^{2}}.$

We propose a bold hypothesis, the class labels of unlabeled data set $x_{u}$ and the class labels of labeled data set have no intersection. Then, we apply the sparse autoencoder algorithm to study a sparse autoencoder from $x_{u}^{\left(1\right)},x_{u}^{\left(2\right)},\ldots ,x_{u}^{\left(k\right)}\in R^{n^{2}}.$ It can be used to rebuild the representation of input training data set $x_{l}^{\left(1\right)},x_{l}^{\left(2\right)},\ldots ,x_{l}^{\left(m\right)}\in R^{n^{2}}$, converting it into a new labeled training set $\left\{{\hat{x}}_{l}^{\left(1\right)},{\hat{x}}_{l}^{\left(2\right)},\ldots ,{\hat{x}}_{l}^{\left(m\right)}\right\}$. These activations are put into the classifier as the new input feature vector, and PLSDA and RBF are employed as classifiers in this paper.

### 3.1. Sparse Autoencoder

#### 3.1.1. Neural Network

The sparse autoencoder algorithm is an unsupervised learning algorithm that applies back-propagation, which is widely applied to image identification [20,26,27].

**Figure 4:**
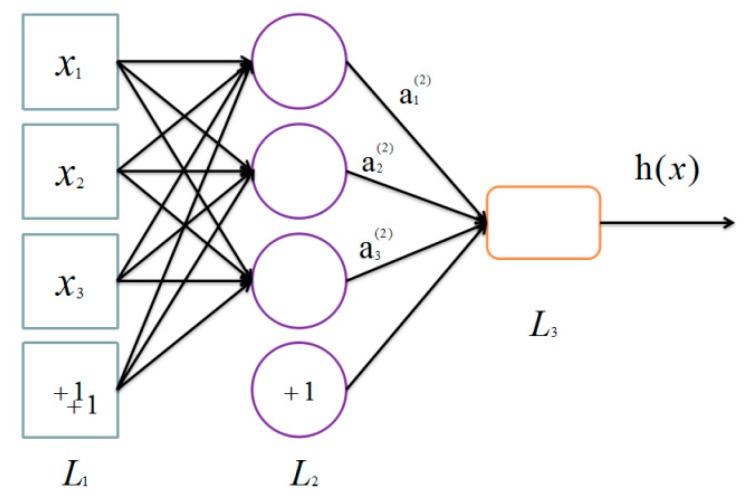
Basic neural network.

A single-layer autoencoder [28,29] is a kind of neural network [30,31,32] that only has one hidden layer. By hooking together many simple “neurons”, a neural network is created. In this paper, each $x_{u}$ is a neuron. For example, here is a small neural network (Figure 4).

Then, there is a way of defining a complex non-linear form of hypotheses h(x), with parameters *W*, *b* that can fit our data. Formally, corresponded to an input $x_{u}\in R^{n^{2}}$, the activations of $x_{u}$ are

(1) $$a_{1}^{\left(2\right)}=f\left(W_{11}^{\left(1\right)}x_{1}+W_{12}^{\left(1\right)}x_{2}+W_{13}^{\left(1\right)}x_{3}+b_{1}^{\left(1\right)}\right),\;a_{2}^{\left(2\right)}=f\left(W_{21}^{\left(1\right)}x_{1}+W_{22}^{\left(1\right)}x_{2}+W_{23}^{\left(1\right)}x_{3}+b_{2}^{\left(1\right)}\right),\;a_{3}^{\left(2\right)}=f\left(W_{31}^{\left(1\right)}x_{1}+W_{32}^{\left(1\right)}x_{2}+W_{33}^{\left(1\right)}x_{3}+b_{3}^{\left(1\right)}\right),\;h\left(x\right)=a_{1}^{\left(3\right)}=f\left(W_{11}^{\left(2\right)}a_{1}^{\left(2\right)}+W_{12}^{\left(2\right)}a_{2}^{\left(2\right)}+W_{13}^{\left(2\right)}a_{3}^{\left(2\right)}+b_{1}^{\left(2\right)}\right),$$

where we define f(·) to be the sigmoid function $f\left(z\right)=\frac{1}{1+\exp\left(-z\right)}$, and $a_{i}^{\left(l\right)}$ denotes the activation (output value) of unit i in layer l, $b_{i}^{\left(l\right)}$ represents the bias associated with unit i in layer l , $\;W_{ij}^{\left(l\right)}$ is the parameter (or weight) associated with the connection between unit j in layer l, and unit i in layer l+1.

We can write these equations more compactly as

(2) $$a^{\left(l\right)}=f\left(W^{\left(l-1\right)}x+b^{\left(l-1\right)}\right).$$

In this sequel, $a^{\left(l\right)}$ is the vector of activations of layer l, $W^{\left(l-1\right)}$ is the weight matrix of$\;a^{\left(l\right)}$, and similarly $b^{\left(l-1\right)}$ is the bias vector that computes $a^{\left(l\right)}$.

More generally, we define a equation that

(3) $$z_{i}^{\left(l\right)}=\sum_{j=1}^{n}W_{ij}^{\left(l-1\right)}a_{j}^{\left(l-1\right)}+b_{i}^{\left(l-1\right)}.$$

Therefore we have$\;a_{i}^{\left(l\right)}=f\left(z_{i}^{\left(l\right)}\right)$. For a neural network which owes p layers, we have output$\;h\left(x\right)=a^{\left(p\right)}$, and we call the process to compute$\;a^{\left(l\right)}$ from $a^{\left(1\right)}$ to$\;a^{\left(p\right)}$ (h(x)) as “the feedforward pass”.

After performing a feedforward pass, we initialize $W_{ij}^{\left(l\right)}$ and $b_{i}^{\left(l\right)}$ to the value near 0 nearly, then the gradient descent algorithm incorporated with BP (backpropagation) is employed as an optimization algorithm.

The cost function is defined as follows:

There is $J\left(W,b;x,y\right)=\frac{1}{2}{\left||h\left(x\right)-y|\right|}^{2}$ for each sample, and because we have k unlabeled samples, thus the overall cost function can be written as:

(4) $$\begin{array}{cc} J\left(W,b\right) & =\left[\frac{1}{k}\sum_{i=1}^{k}J\left(W,b;x^{\left(i\right)},y^{\left(i\right)}\right)\right]+\frac{\mu }{2}\sum_{l=1}^{p-1}\sum_{i=1}^{s_{l}}\sum_{j=1}^{s_{l}+1}{\left(W_{ji}^{\left(l\right)}\right)}^{2},\\ & =\left[\frac{1}{k}\sum_{i=1}^{k}(\frac{1}{2}{\left||h\left(x\right)-y|\right|}^{2})\right]+\frac{\mu }{2}\sum_{l=1}^{p-1}\sum_{i=1}^{s_{l}}\sum_{j=1}^{s_{l}+1}{\left(W_{ji}^{\left(l\right)}\right)}^{2} \end{array}$$

where μ controls the relative importance of the two terms. In our project, we set μ as 3 × 10^−3^. Our target is to minimize J(W,b), here we repeatedly implement the batch gradient descent to reduce our cost function J(W,b). One iteration of batch gradient descent is shown as follows:

1. Make $\Delta W^{\left(l\right)}:=0,\;\Delta b^{\left(l\right)}:=0$ (matrix/vector of zeros) for all l
2. For i = 1 to k, we use BP to compute $\nabla_{W^{\left(l\right)}}\;J\left(W,b;x,y\right)$ and $\nabla_{b^{\left(l\right)}}\;J\left(W,b;x,y\right)$ firstly, then set
  (5) $$\begin{array}{r} \Delta W^{\left(l\right)}:=\Delta W^{\left(l\right)}+\nabla_{W^{\left(l\right)}}\;J\left(W,b;x,y\right),\;\\ \Delta b^{\left(l\right)}:=\Delta b^{\left(l\right)}+\nabla_{b^{\left(l\right)}}\;J\left(W,b;x,y\right). \end{array}$$
3. Finally, we transform our parameters as:
  (6) $$\begin{array}{lll} W^{\left(l\right)} & := & W^{\left(l\right)}-\alpha \left[\frac{1}{k}\Delta W^{\left(l\right)}+\mu W^{\left(l\right)}\right],\;\\ b^{\left(l\right)} & := & b^{\left(l\right)}-\;\alpha \left[\frac{1}{k}\Delta b^{\left(l\right)}\right], \end{array}$$
  where α is the learning rate. In step 2, it is crucial to compute the partial derivatives via BP, which is detailed described as following:
  I For each output unit i in layer p (the output layer), set
    (7) $$\delta^{\left(p\right)}=-\left(y-a^{\left(p\right)}\right)\cdot f^{\prime }\left(z^{\left(p\right)}\right),$$
    where $\delta^{\left(p\right)}$ is defined as the difference between the network’s activation and the true target value.
  II For l=p−1,p−2,…,2, set
    (8) $$\begin{array}{r} \delta^{\left(l\right)}=\left({\left(W^{\left(l\right)}\right)}^{T}\delta^{\left(l+1\right)}\right)\cdot f^{\prime }\left(z^{\left(l\right)}\right),\\ f^{\prime }\;\left(z^{\left(l\right)}\right)=a^{\left(l\right)}\left(1-a^{\left(l\right)}\right). \end{array}$$
  III Compute the partial derivatives
    (9) $$\begin{array}{r} \nabla_{W^{\left(l\right)}}\;J\left(W,b;x,y\right)=\delta^{\left(l+1\right)}{\left(a^{\left(l\right)}\right)}^{T},\\ \nabla_{b^{\left(l\right)}}\;J\left(W,b;x,y\right)=\delta^{\left(l+1\right)}. \end{array}$$

Notably, BP is not that easy to debug and get right. In the following section, we provide a derivative checking procedure to check the correctness of the code and make sure our implementing of gradient descent is correct. In a correct code we have:

(10) $$\nabla_{W^{\left(l\right)}}\;J\left(W,b\right)=\frac{1}{k}\Delta W^{\left(l\right)}+\mu W^{\left(l\right)},\;\nabla_{b^{\left(l\right)}}\;J\left(W,b\right)=\frac{1}{k}\Delta b^{\left(l\right)}.$$

If the equation is satisfied, it proves that we indeed get the correct derivations. In practice, we define θ as a vector unrolling the parameters W, b. Thus, when a function $g\left(\theta \right)=\frac{dJ\left(\theta \right)}{d\theta }$ is given, we can verify its correctness by checking that whether the following formula is satisfied.

(11) $$g\left(\theta \right)\approx \frac{J\left(\theta +\epsilon \right)-J\left(\theta -\epsilon \right)}{2\epsilon }.$$

In practice, we set ϵ which is always around 10^−4^ to a small constant.

#### 3.1.2. Autoencoders and Sparsity

Thus far, we have described the application of neural network to supervised learning, but we have only used the unlabeled training data set; an autoencoder neural network that combines BP is introduced to deal with such a situation.

The auto encoder tries to learn an identity function that enforces h(x)≈x, which means the target value is $y_{u}^{\left(i\right)}=x_{u}^{\left(i\right)}$.

**Figure 5:**
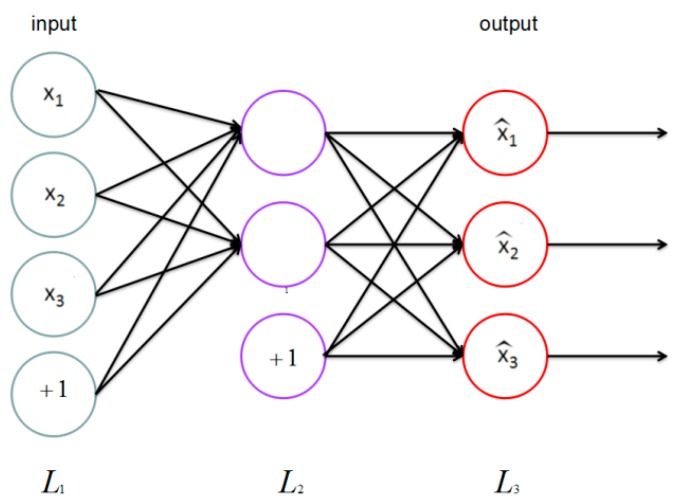
Sparse autoencoder.

As Figure 5 shows, this is a simple auto encoder. Our goal is to enforce output $\hat{x}$ to be similar to input x. To achieve this, we set constraints on the ordinary neural network.

We let $a_{j}^{\left(2\right)}\left(x\right)$ denote the activation of this hidden unit when the network is given a specific input x. And next step we compute the average activation of hidden unit j.

(12) $$\hat{\rho_{J}}=\frac{1}{k}\overset{\;k}{\underset{i=1}{\sum }}\left[a_{j}^{\left(2\right)}\left(x^{\left(i\right)}\right)\right].$$

We impose a constraint to$\;\hat{\rho_{J}}$ that $\hat{\rho_{J}}=\rho ,$ where ρ is a sparsity parameter whose value is close to zero. In our paper, we set it as 0.01. j is the sum of whole hidden units in the network.

Performing the above two equations, we are ready to complete the overall cost function on the basis of Equation (4) to be

(13) $$J_{sparse}\left(W,b\right)=J\left(W,b\right)+\beta \overset{s_{2}}{\underset{j=1}{\sum }}KL(\rho ||\hat{\rho_{J}}),$$

where J(W,b) is the same as the original one, β is set as 3 in our project, controlling the weight of the sparsity penalty term.${\;s}_{2}$ denotes the number of nodes in the hidden layer; and as Equation (13) shows, we apply KL-divergence as the penalty term, which can be expressed as $KL(\rho ||\hat{\rho_{J}})=\rho log\frac{\rho }{\hat{\rho_{J}}}+\left(1-\rho \right)log\frac{1-\rho }{1-\hat{\rho_{J}}}.$ Until now, the KL-divergence term has been adopted to satisfy the constraints, also, to integrate the KL-divergence into our derivative calculation, we adjust Equation (8):

(14) $$\delta^{\left(2\right)}=\left(\left({\left(W^{\left(2\right)}\right)}^{T}\delta^{\left(3\right)}\right)+\beta \left(-\frac{\rho }{\hat{\rho_{J}}}+\frac{1-\rho }{1-\hat{\rho_{J}}}\right)\right)\cdot f^{\prime }\left(z^{\left(2\right)}\right).$$

Other steps are the same as the neural network algorithm, and we perform a gradient descent on the new objective $J_{sparse}\left(W,b\right)$. Still we apply the derivative checking method to verify our code. The algorithm, therefore, encourages the activations $a_{u}$ to be sparse, in other words, for most of its elements to be zero. At the same time we learn the basis vector θ (unrolling parameters W,b into a long vector). It is the basis of both $x_{u}$ and $x_{l}$.

### 3.2. Construct New Representation

So far, we have trained a basis vector θ (a set of parameters $W^{\left(1\right)},\;W^{\left(2\right)},\;b^{\left(1\right)},\;\;b^{\left(2\right)}$), which would be used to construct the new labeled data set $\left\{{\hat{x}}_{l}^{\left(1\right)},{\hat{x}}_{l}^{\left(2\right)},\ldots ,{\hat{x}}_{l}^{\left(m\right)}\right\}$ based on original labeled data set. We pose the following formulation to solve the problem, for each$\;x_{l}^{\left(i\right)}$, we have

(15) $${\hat{x}}_{l}^{\left(i\right)}=f\left(W^{\left(2\right)}f\left(W^{\left(1\right)}x_{l}^{\left(i\right)}+b^{\left(1\right)}\right)+b^{\left(2\right)}\right),$$

**Figure 6:**
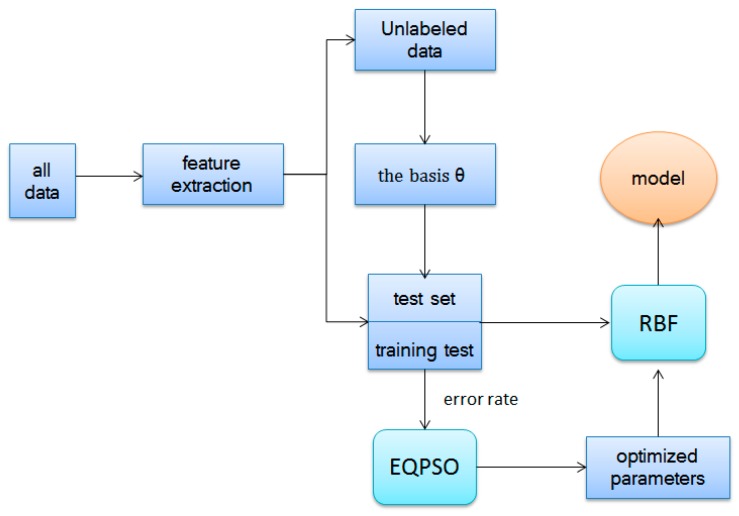
Flow chart of self-taught learning.

The reconstructing procedure decreases the difference between the labeled data and unlabeled data as well as transfers knowledge from different domains. These new features are put into multiple types of classifications, such as RBF. The whole algorithm of self-taught learning based on sparse autoencoder is illustrated in Algorithm 1 and Figure 6.

**Algorithm 1. Self-taught learning algorithm****Step 1**: Minimize $J_{sparse}\left(W,b\right),$ train the basis vector θ (unrolling parameters W,b into a long vector) from unlabeled data$\;x_{u}$.**Step 2**: Take advantage θ to construct new representation$\;{\hat{x}}_{l}$, replacing original data set $x_{l}$.**Step 3**: Learn a classifier by applying efficient algorithm (we apply RBF and PLSDA optimized by EQPSO here).**Step 4**: Calculate the classification accuracy.

## 4. Results and Discussion

To verify the feasibility of the model we performed some experiments and obtained some results, which will be shown in this section. In order to improve the performance of the classifier, EQPSO is applied to optimize the parameters, both for RBF and PLSDA.

In all experiments, the particle number of optimization algorithm is set to 30, and the algorithm iterates 300 times to find the optimal value. Additionally, we set the hidden layer as 10 at first, the sparsity parameter as 0.01, β as 3 in this project to get a sparse autoencoder. All the data set, which is the input of classifier, has been normalized before self-taught learning.

**Figure 7:**
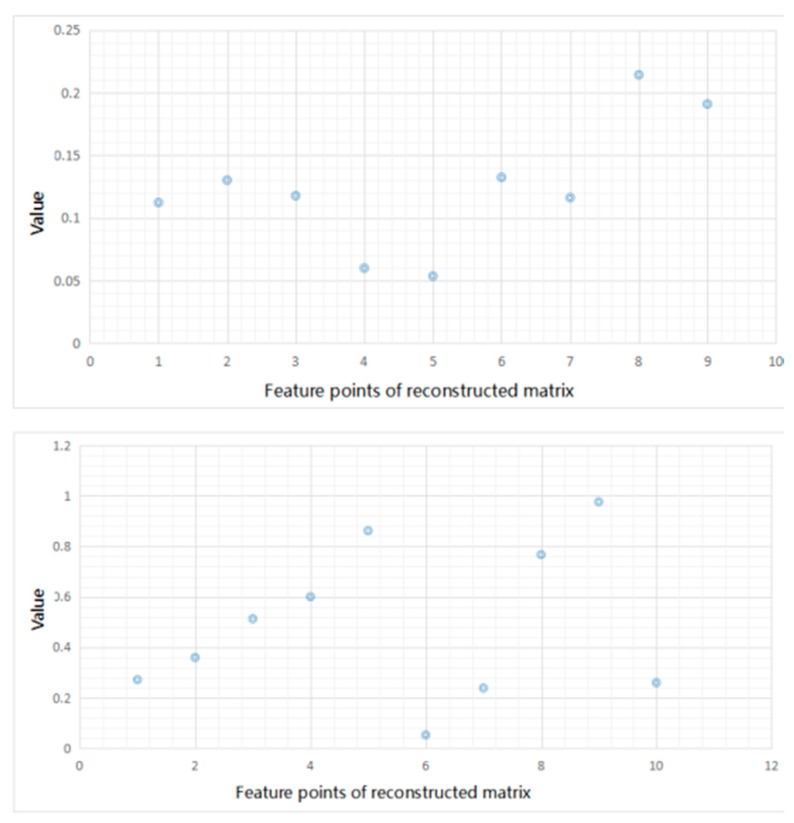
Input feature matrix and reconstructed matrix of one sample.

Before showing the results, we display the input feature matrix and the reconstructed ones in Figure 7. We take one sample of *S. aureus* as an example. In this experiment, the hidden layer is set as 10 and the dimension of input matrix is 3 × 3. The left picture of Figure 7 shows the feature matrix of input matrix and the right one shows the reconstructed matrix.

Firstly, considering that each sensor has a different selectivity pattern, the performances could change without increasing the size of the matrix, but only selecting the best subset of features. So we combine different sensors with different features from dimension 3 × 3 to 5 × 5 and finally find that the results are almost the same when dimensions are the same. Then we discuss whether the self-taught learning could improve the performance of the E-nose and how the dimension of feature matrix influences the classification accuracy. We apply RBF as the classifier here and 15 samples of each wound infection serve as the training data, and 5 samples of each wound infections are the test data. The test set is used to verify the performance of the final model. In Table 5, we show the results studied from 652 gas samples.

**Figure 8:**
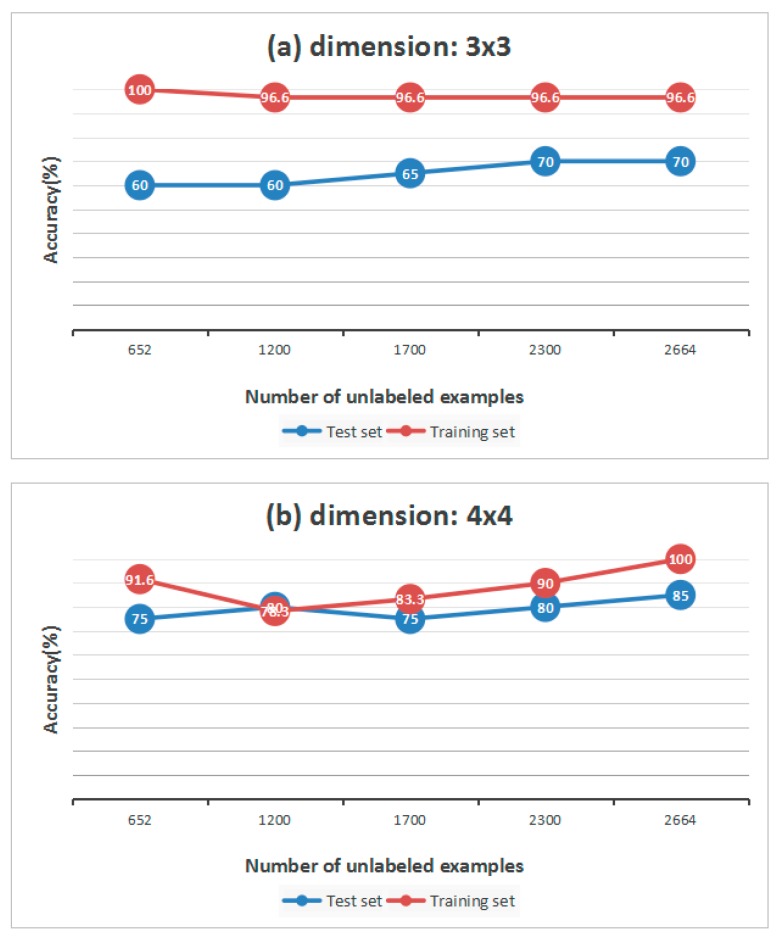
Accuracy with different number of unlabeled samples. (**a**) shows the change of accuracy as the number of examples increases when dimension is 3 × 3; (**b**) shows the change of accuracy as the number of examples increases when dimension is 4 × 4.

In Table 5, we find that, compared to the raw matrix, from dimension 3 × 3 to 5 × 5, the matrix studied from unlabeled samples has higher accuracy overall. However, when the dimension is 3 × 3, the accuracy of the training set and test set do not change much, which means that if the dimension is small, the accuracy may not improve. At the same time, however, the number of unlabeled samples also contributes to the results of classification. We change the size of unlabeled data set from 652 to 2664 to explore the relationship between them. All of the results are shown in Figure 8.

In general we can draw a conclusion that, the more unlabeled samples, the higher the accuracy. If the interval of two sizes is small, the accuracy may not change. When the dimension is 3 × 3, the classification accuracy of the test set keeps rising, while in Figure 8b, the curve of the training set goes down when the size of unlabeled data set is 1200, and the curve of the test set goes down when the size of the unlabeled data set is 1700.

It is well established that the classifier is a major part of the E-nose; thus, PLSDA and RBF are introduced to distinguish wound infection samples respectively in our paper. In order to compare the differences in putting the feature matrix into these two classifiers directly and the representations processed by self-taught learning, we calculate the accuracy of both. The accuracy is shown in Table 6.

**Figure 9:**
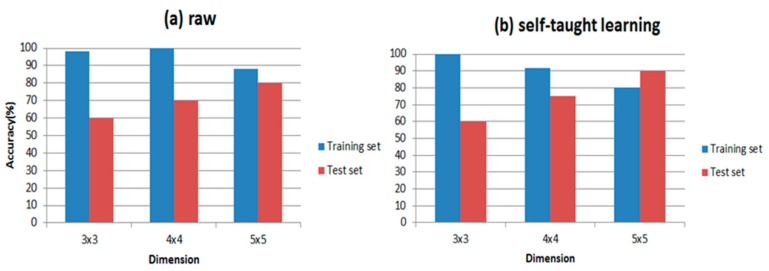
Accuracy with RBF. When dimension changes from 3 × 3 to 5 × 5, (**a**) shows the accuracy of unprocessed data set (training set and test set); (**b**) shows the accuracy of data set processed by self taught learning.

**Figure 10:**
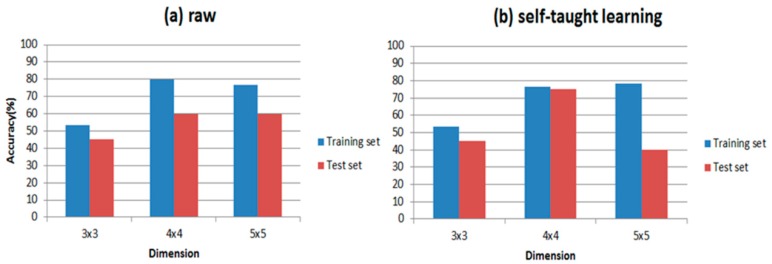
Accuracy with PLSDA. When dimension changes from 3 × 3 to 5 × 5, (**a**) shows the accuracy of unprocessed data set (training set and test set); (**b**) shows the accuracy of data set processed by self taught learning.

Table 6 shows the results of our experiments and the classification accuracies are clearly presented in it. Comparing Figure 9 with Figure 10, RBF is indeed more suitable for self-taught learning than PLSDA, because all of the accuracies of RBF are higher than accuracies of PLSDA, especially for the test data set. Additionally, comparing (a) with (b) in Figure 9 and Figure 10, the accuracy of self-taught learning rises more quickly than the raw matrix. As for PLSDA, the results are not very good and steady, when the input is raw matrix, the accuracy of test data grows slowly. All results in Figure 9 and Figure 10 prove that RBF is superior to PLSDA in self-taught learning based on the sparse autoencoder. In Figure 10b, when the input is studied from the unlabeled data set, the accuracy of the test set reaches its peak at 4 × 4, then the curve falls down to 40% at 5 × 5.

All experiments above are established under the condition that the hidden layer is ten. In order to study the difference that the hidden layer brings to the accuracy, we set the hidden layer at 5, 10, 20, 40, 100, 700, 2000, 10,000.

Except for hidden layer, all the other parameters remain the same. On account of the instability of the classification accuracy, to make sure the result of the experiment is correct, each program is repeated 5 times.

Table 7, Table 8 and Table 9, respectively, show the classification accuracy with different hidden layers when the dimension is 5 × 5, 4 × 4 and 3 × 3. From Table 7, Table 8 and Table 9, we can draw a conclusion that as the hidden layer increases, the classification accuracy always reaches the top at first and falls down later. And when the hidden layer is large, the accuracy always maintains at a certain level; at the same time, it takes more time to train the model, which means increasing the hidden layer can lead to a waste of time.

In conclusion, these results prove that the self-taught learning based on sparse autoencoder demonstrates a good performance in improving the accuracy by studying samples from other fields, and that there are a few reasons for why this is possible with the sparse autoencoder. Firstly, as a typical algorithm of traditional training multi-layer network, the BP algorithm is not ideal for only a few layers of network. If all layers are trained at the same time, the time complexity will be too high; If only one layer is trained, the bias will pass by the layer. This will face the opposite problem of the above supervised learning, which will be badly mismatched. Self-taught learning based on sparse autoencoder is a kind of layer-wise-pre-training, which solves the problem effectively. Furthermore, the sparsity constraint makes the representation of each layer sparse (most of nodes become zero). This kind of representation resembles the human brain—when something comes to our mind, only a small number of neurons are stimulated and other neurons are suppressed. This feature is the same with humans when we want to study new things from other fields.

## 5. Conclusions

The self-taught learning approach is a new model of transfer learning and has not been used in E-nose before; in this paper, we have applied self-taught learning to wound infections classification.

In this paper we introduce a kind of self-taught learning based on a sparse autoencoder to the E-nose in wound infection detection, and we take advantage of PLSDA and RBF which are optimized by EQPSO to classify four kinds of wound infections. Through comparing the results of self-taught learning and the results of the raw data set, we can draw a conclusion that the performance rises when we apply self-taught learning based on sparse autoencoder, especially with the RBF classifier. We also found that the size of unlabeled data set, the type of classifier and the dimensions of data set all have an impact on the accuracy of pattern-recognition.

These results prove that the self-taught learning based on sparse autoencoder has a good performance in transforming knowledge, and indeed, could improve the accuracy by studying samples from other fields. However, it still has its limits. First of all, when the number of unlabeled samples is small, especially when the order of magnitude is smaller than 10, the algorithm barely improves the performance.

Through self-taught learning, we can reduce costs when we train the E-nose for distinguishing wound infections, which is the purpose of our experiments. We all know that the data of such gas is not easy to obtain, thus we study knowledge from unlabeled data set in other fields, which means that we can have a significant amount of data to study, making up for the lack of wound infection samples and transferring it to the area of wound infections.

In future work, we will further study the self-taught learning applied in E-nose, and we believe E-nose will be further improved in the field of medical science.

## Figures and Tables

**Table 1:** Sensitive characteristics of gas sensors.

Sensors	Sensitive Characteristics
TGS813	Methane, Propane, Ethanol, Isobutane, Hydrogen, Carbon monoxide
TGS816	Combustible gases, Methane, Propane, Butane, Carbon monoxide, Hydrogen, Ethanol, Isobutane
TGS822	Organic solvent vapors, Methane, Carbon monoxide, Isobutane, n-Hexane, Benzene, Ethanol, Acetone
TGS2600	Gaseous air contaminants, Methane, Carbon monoxide, Isobutane, Ethanol, Hydrogen
MQ135	Ammonia, Benzene series material, Acetone, Carbon monoxide, Ethanol, Smoke

Note: The response of these three sensors is non-specific. Table 1 just lists their main sensitive gases, and they are also sensitive to other gas.

**Table 2:** Pathogens in wound infection and their metabolites.

Pathogens	Metabolites
*S. aureus*	Acetic acid, Aminoacetophenone, Ammonia, Ethanol, Formaldehyde, Isobutanol, Isopentyl acetate, Isopentanol, Methyl ketones, Trimethylamine, 1-Undecene, 2,5-Dimethylpyrazine isoamylamine, 2-Methylamine
*E. coli*	Acetaldehyde, Acetic acid, Aminoacetophenone, Butanediol, Decanol, Dimethyldisulfide, Dimethyltrisulfide, Dodecanol, Ethanol, Formaldehyde, Formic acid, Hydrogen sulfide, Indole, Lactic acid, Methanethiol, Methyl ketones, Octanol, Pentanols, Succinic acid, 1-Propanol
*P. aeruginosa*	Butanol, Dimethyldisulfide, Dimethyltrisulfide, Esters, Methyl ketones, Isobutanol, Isopentanol, Isopentyl acetate, Pyruvate, Sulphur compounds, Toluene, 1-Undecene, 2-Aminoacetophenone, 2-Butanone, 2-Heptanone, 2-Nonanone, 2-Undecanone

**Table 3:** Concentration of the target gases.

Gases	Concentration Range (ppm)	Number of Samples
benzene	[0.1721, 0.7056]	480 (12 × 12)
formaldehyde	[0.0668, 0.1425]	491 (12 × 11)
acetone	[0.0565, 1.2856]	549 (12 × 12)
ethylalcohol	[0.0832, 0.6732]	1144 (12 × 21)

**Table 4:** Amount of samples.

Pollutant Gas	Amount of Samples	Wound Infection	Amount of Samples
benzene	132	*S. aureus*	20
formaldehyde	203	*E. coli*	20
acetone	153	*P. aeruginosa*	20
ethylalcohol	164	uninfected	20

**Table 5:** Accuracy of three kinds of feature matrix with radial basis function (RBF) (%).

Dimension	Raw		Spares Autoencoder	
	Training Set	Test Set	Training Set	Test Set
3 × 3	98.3	60	100	60
4 × 4	100	70	91.6	75
5 × 5	88.3	80	80	90

**Table 6:** Accuracy with partial least squares discriminant analysis (PLSDA) and RBF (%).

Dimension		RBF		PLSDA	
		Train Set	Test Set	Train Set	Test Set
3 × 3	raw	98.3	60	53.3	45
	sa	100	60	53.3	45
4 × 4	raw	100	70	80	60
	sa	91.6	75	76.6	75
5 × 5	raw	88.3	80	76.6	60
	sa	80	90	78.3	40

Note: “sa” in Table 6 represents “spares autoencoder”.

**Table 7:** Accuracy with different hidden layer (%).

Hidden Layer	5	10	20	40	100	700	2000	10,000
Training set	96.6	80	93.3	84.15	91.6	91.6	91.6	91.6
Test set	65	90	90	87.5	80	75	75	75

Note: the dimension is 5 × 5 and the classifier is RBF.

**Table 8:** Accuracy with different hidden layer (%).

Hidden Layer	5	10	20	40	100	700	2000	10,000
Training set	88.3	91.6	100	89.15	100	91.6	95	100
Test set	80	75	75	80	75	85	80	70

Note: the dimension is 4 × 4 and the classifier is RBF.

**Table 9:** Accuracy with different hidden layer (%)

Hidden Layer	5	10	20	40	100	700	2000	10,000
Training set	96.6	100	100	98.3	100	96.6	98.3	98.3
Test set	60	60	60	75	65	75	70	65

Note: the dimension is 3 × 3 and the classifier is RBF.
